# Occupational Care Giving Conditions and Human Rights: A Study of Elderly Caregivers in Botswana

**DOI:** 10.4103/0973-1075.68409

**Published:** 2010

**Authors:** Simon Kangethe

**Affiliations:** Department of Adult Education Centre For Continuous Education University of Botswana P/B UB 00707 Gaborone, Botswana

**Keywords:** Elderly caregivers, Occupational care giving, Human rights, PLWHA: Orphans

## Abstract

The article aims to explore and discuss the occupational care giving conditions pitting them against human rights. The article’s objective is to initiate discussions and generate literature pertaining to occupational care giving load and assessing the human rights challenge it poses. The article uses analysis of the literature review from an array of eclectic data sources. The following factors were found besetting the caregivers’ human rights: (1) Aging; (2) Cultural and community attitudes towards care giving; (3) Risk of contagion; (4) Health hazards and lack of compensation. Recommendations: (1) Adoption of grandparents/grandchildren care symbiosis system; (2) Government remuneration policy for caregivers; (3) Mainstreaming of gender education to encourage men and youth develop an interest in care giving; (4) Institution of laws and policies by countries to provide for the compensation of caregivers’ occupational hazards and risks.

## PROBLEM STATEMENT

Raising awareness of caregivers’ occupational risks and hazards and relating them to human rights context is pertinent and central. This is because of the apparent perception that caregivers, as opposed to their clients (acquired immune deficiency syndrome clients or orphans), have been taken for granted and yet they have been performing sterling and invaluable tasks of mitigating the AIDS pandemic through caring for the AIDS clients and orphans. Apparently, the policy and operationalization gaps pertaining to their assistance have glaring lacunae in countries such as Botswana. This paper, therefore, is a platform to raise the bell to policy makers and care managers in the HIV/AIDS and orphan field, to robustly consider looking into those prevalent gaps.

The human rights phenomenon pertaining to caregivers has also been found wanting. Apparently, while other health service providers in many countries have their occupational risks and hazards well taken care of in terms of compensation, the case for the caregivers is alarming and has not attracted any occupational health compensation agenda. This paper, therefore, acts as an advocacy and lobbying platform to advocate for a policy environment that would put the compensational agenda for the caregivers on the same footing as other health service providers. All the human rights pertaining to care and humane treatment need to be taken on board.

## INTRODUCTION AND BACKGROUND

Care giving occupations should attract health rights just like any other health occupation. Kangethe[1] noted that while the care programs of people living with HIV/AIDS in Botswana attract elegant policies and considerations, those of the caregivers are neither well developed nor operationalized. This also casts doubt as to the realization of the caregivers’ occupational health rights. According to the Human Development Report,[2] health and human rights are inextricably linked. Healthcare is richly grounded in the Universal Declaration of Human Right that says: “Everyone has the right to a standard of living adequate for his/her own health and well being of his/her family, including food, clothing, housing and medical care and other necessary social services.”[3] It is inadequate availability and access to most of the care giving requisite services that immensely point to the glaring gaps and caregivers’ human rights violation.[1]

Today, it is glaringly an incontrovertible fact that all governments globally are unconditionally obliged to avail of and access these health rights to its citizenry. It is also a step towards achieving most of the health goals and universal access as spelt out in the Global Millennium Development Goals.[4] Access to health and care as human rights is not a privilege, but a universal, conventional and imminent obligation that the governments are supposed not only to safeguard, but also holistically provide to its people.[2,5] Despite this imperative, many governments have been resource- constrained, necessitating that communities lend a hand to their governments in order to mitigate the challenge.[6] This has made many governments integrate community home-based care structures into the mainstream healthcare systems, with the country of Botswana leading the pack.[7] It is due to this scenario that many elderly people, especially women, have unfairly been at the brunt edge of care, which is sometimes too heavy for them and posing to drive them fast to the graves.[1] Though in some exceptional cases, the elderly are invaluable asset to the care business, in some cases, it is proving too difficult, cumbersome, abusive and sometimes suicidal. This is when the caregivers’ conditions of work and their human rights are given a raw deal.[8]

In his 2005-2006 research in Botswana, this author was in pain when he went to collect an 85-year-old caregiver and found her struggling to push a trolley carrying her brother to the direction of the tree shade. It was a painstaking experience for an elderly mother who really required to be taken care of instead of taking care of somebody. Whatever be the circumstances that pushed this elderly grandmother to take care of her brother who had suffered a stroke, it was definitely a gross violation of her human rights. The task was socially, physically, psychologically and emotionally draining. This author had to agree with this caregiver and others in the focus group discussion that care giving was likely to drive some caregivers fast into the graves. Aging is also known to go hand-in-hand with constant sickness.[8,9] Psychologists do acknowledge the fact that the change towards senility is also marked by a decline in cognitive, emotional and physical energies affecting one’s productivity holistically. Aging is also believed to follow the second law of thermodynamics principle, which maintains that entropy of the universe is constantly increasing because of the dissipation of energy from system to system.[8,10] Subjecting the elderly to care giving load is indeed a human rights abuse as it inconveniences their healthy well being.[2]

## FACTORS VIOLATING ELDERLY CAREGIVERS’ RIGHTS

### Aging

Care by the elderly poses serious challenges as the caregivers experience a state of stress, anxiousness and dilemma concerning the fate of their clients (either an AIDS client or an orphan) upon their death. This is apparent in Zimbabwe where irinews[11] gives an account of a caregiver, Ndanda Ncube who, at the age of 80 should be settling into retirement, but the HIV/AIDS pandemic has brought a new burden of responsibilities for her. She has to wake up in the morning to do the household chores, gather firewood and feed her six grandchildren. At this age, this is unfair, abusive and stretches her human rights from taking rest and instead takes tasks that stretch her social, physical, psychological and emotional capacities beyond limit.[12]

Ndanda Ncube is reportedly fearing and expressing her anxiety by echoing her feelings: “I am old and soon I will move on. Who will look after the children and provide for them?” This leaves unanswered questions and is an indicator of grief and uncertainty of the future for her clients. This raises the importance of counseling to help “recharge batteries” to energize her working capacities.[13]

### Community attitudinal change towards care giving

It is a stark naked fact that the situation of care giving as a safety net appears to be weakening as extended families succumb to nuclear family systems, especially with the advent and increased pace of eurocentricim, westernization, modernization and globalization.[1,12] African brotherhood and the spirit of caring, especially for the distressed members of the communities that Mensah[14] had envisaged has been under attack from all sides by these forces of modernization. This has seen more burden on the individual caregiver as community responsibility over the cared individuals shifts the gear.

This researcher’s 2005-2006[1] study findings in Kanye in Botswana empirically confirm and present the same version of desperation of care bestowed on the shoulders of the elderly. They had the following sentiments to make:

“some of us are old and we need to be cared for, not to care”

“Some of us due to our age, we sometimes get confused by care giving. We do not even know the results and quality of our care giving”

“Relatives and family members help only a little”

This is an indicator of how the quality of care in Botswana may be compromised on. This should send signals to the governments and policy makers to investigate further the quality of care giving and also the human resource available to run and effect community home based care programs on the ground.

### Risk of contagion

The phenomenon of contracting HIV/AIDS by the elderly presents a worrying state of affairs. This is because in their age, understanding the viral epidemiology adequately is a challenge. In his Kanye research study, Kangethe[1] was told by some elderly caregivers that they only understood well when their clients were improving but found it difficult to track the disease progression of their clients, and that some caregivers could have succumbed to the disease.[15] This further indicates their challenge and doubts their capacity as far as understanding issues of hygiene and handling clinical waste facilities are concerned. This leaves a glaring lacuna that could jeopardize caregivers’ health through contracting the disease of their clients. This indeed presents a serious human rights violation.

### Health hazards and compensation

The rights of the elderly caregivers in the face of the occupational hazards and risks need to be debated alongside the rights of other service providers. The Worker’s Compensation Act no 23 of 2001 of the laws of Botswana provides for the compensation of workers for injuries sustained or occupational hazards arising in the course of employment and / or for death resulting from such injuries or occupational diseases. The Act obliges all employers to ensure safety and secure all workers. The employer is also obliged, under Section 11 of the Workers Compensation Act to pay compensation for any such injuries/ death resulting from serving clients.[16] Perhaps it is at this juncture that we need to ask ourselves whether in the event that it is proved that a caregiver succumbs to AIDS through care giving, what would be the position of the government as far as compensation is concerned. This issue is also important because what may be happening on the ground may be violating the human rights of the elderly informal caregivers with nobody taking the platform to advocate for the redress.

## STRATEGIES TO REDRESS ELDERLY CAREGIVING HUMAN RIGHTS PHENOMENON

### Adopt grandparent/grandchildren care symbiosis

It is recommended that the grandparents/grandchildren phenomenon could work under the current resource constrained community situation in developing countries under which HIV/AIDS has driven communities into.[6] As the grandparents struggle to support their grandchildren, especially by being mentors and advisors, the children could also stretch their caring hands by providing labor as a means of production and survival. This system in Africa would exploit the social capital of trust and respect that the children accord the elderly. Though Conventions on the Rights of Children[17] would call this child labor, and indicate that it contravenes Article 15 of the CRC charter, it does not make sense, especially in some African systems in which political, economic and social systems are not tenable, where everyone struggles just to secure a meal for the day struggling to sink or float.[17] Stephen Lewis, former United Nations (UN) special envoy for HIV/AIDS in Africa is in support of this arrangement and qualitatively asserts that ‘grandmother phenomenon’ is a legitimate extended family arrangement and the kids, by and large, are related to one another and they are happy in that sense”[11]

### Government remuneration policy for caregivers

Since most care giving environments in the developing world are poverty laden and, therefore, likely to be distressful, taxing and sometimes with burnout,[18] an attractive remuneration policy and package could reduce the financial and material resource gaps. Botswana does not have a caregiver remuneration policy. Other than natural motivation emanating from blood relationship and kinship ties (*Kangethe paradigm*), this author found no form of motivation, incentive, reward or any strategy to motivate the caregivers. An incentive or reward is known to facilitate positive changes and increased productivity.

While Lawler[19] emphasizes and considers the level of pay as an instrument to measure work morale and therefore expected productivity, Kohn[20] supports the reward system not only from pay, but also in praise or prizes. The government of Botswana, for instance, should borrow a leaf from the Mozambique government that has drawn an attractive policy to remunerate the caregivers with a package of 60% of the government minimum wage, which is about $55 per month.[11] This is adequate goodwill as the country of Botswana is better endowed with financial resources compared to the country of Mozambique.

### Mainstream gender education for men and youth to increase interest in care giving

The preponderance of women, especially elderly women, in many developing countries is a recipe of cultures interwoven within patriarchal power dynamics and gender differential quotients that dictate the place and role of women in care giving. These mindsets, stereotypes and attitudes need to be obliterated so that men as well the younger persons could adequately be attracted into care giving and relieve the elderly to rest or do supervisory tasks in care giving. Robust gender mainstreaming of and education needs to be mounted by the government, non governmental organizations (NGOs) and all segments of the civil society. It may take time for the message to be internalized and bring about a paradigm shift effect, but the earlier the onslaught, the better.[21] In Botswana, advocacy and civil society campaign for increased government goodwill to sign the South African Gender Protocol may usher in a new thinking dispensation that is likely to change gender thinking amongst all the Batswana. This is likely to make communities see sense of relieving the elderly, especially women from their caring duties that no doubt violate the human rights associated with health.[22]

### Laws of countries to provide for compensation of caregivers occupational hazards and risks

This author believes it is a denial of informal caregivers’ human rights not to be covered by compensation policies that cover other health service providers against any workplace occupational hazards and risks. This happens in Botswana and other developing countries. The fact that caregivers are not formally employed does not make them any less worthy of the compensation. In fact because of their vulnerability emanating from age, poverty, in exposure, illiteracy, they may be more prone to occupational risks and hazards of care giving. I’m advocating for the laws of different countries to cover for the compensation of caregivers’ risks in the eventuality of a hazard. Workers’ compensation Act No 23 of 2001 of the laws of Botswana that provide for the compensation of workers for injuries sustained or occupational hazards needs to be adjusted so as to cover these informal service providers in the event of any occupational hazard or risks.[17]

## CONCLUSION

Elevating the place of informal caregivers to enjoy all the human rights enshrined in the Universal Declaration of Human Rights is critical and pivotal. Botswana as well as many other African countries are signatory to these global human rights conventions. The environment of the caregiver has to be conducive. The care giving load that compromises her physical, social, psychological and emotional human rights needs to be investigated and redressed. The health rights of the caregiver also need to be taken on board.
